# The current status of non-radiologist-performed abdominal ultrasonography in paediatrics – a scoping literature review protocol

**DOI:** 10.1007/s00247-019-04452-y

**Published:** 2019-08-27

**Authors:** Elsa A. van Wassenaer, Joost G. Daams, Marc A. Benninga, Karen Rosendahl, Bart G. P. Koot, Samuel Stafrace, Owen J. Arthurs, Rick R. van Rijn

**Affiliations:** 1 Department of Paediatric Gastroenterology and Nutrition, Emma Children’s Hospital – Amsterdam UMC, University of Amsterdam, Amsterdam, the Netherlands; 2 Medical library, Amsterdam UMC, University of Amsterdam, Amsterdam, the Netherlands; 3 Department of Radiology, Section of Pediatric Radiology, Haukeland University Hospital, Bergen, Norway; 4 Department of Radiology, Sidra Medical and Research Centre, Doha, Qatar; 5 Department of Radiology, Great Ormond Street Hospital for Children NHS Foundation Trust, London, UK; 6 Department of Radiology, Emma Children’s Hospital – Amsterdam UMC, University of Amsterdam, Meibergdreef 9, 1105 AZ Amsterdam Zuid-Oost, the Netherlands

**Keywords:** Adolescents, Children, Point-of-care ultrasound, Scoping review, Ultrasound

## Abstract

In recent years as a result of decreasing prices and the increasing availability of portable systems, ultrasonography (US), which historically has primarily been the domain of radiologists, has become more widely available to non-radiologists as well. This has increased the use of point-of-care paediatric US performed by non-radiologists. With this scoping review, focused on abdominal imaging, we aim to gain an overview of the current practices in the paediatric setting and to assess its impact in daily practice. We present the background and study design of a scoping review for non-radiologist-performed abdominal point-of-care paediatric US using a formal scoping framework. The information shall be derived from published studies. We will submit the review report to a peer-reviewed scientific journal and explore other scientific venues for presenting the work. Based on the completed review, the officers of the European Society of Paediatric Radiology will issue a position statement on non-radiologist-performed point-of-care paediatric US.

## Introduction

One of the most widely used imaging techniques in paediatric radiology is ultrasonography (US). This is a fast, relatively cheap and radiation-free approach to cross-sectional imaging, making it very suitable for use in the vulnerable paediatric patient population. US can be performed under many circumstances ranging from the high-tech environment of a paediatric university hospital to a Third World outpatient clinic. In recent years a paradigm shift has occurred. US, which historically has primarily been the domain of radiologists, has become more widely available to non-radiologists as well. This is mainly due to the fact that US systems have become cheaper and even portable systems that can work on a smartphone have become available. This has resulted in a much wider use of US at the level of primary point-of-care providers and inclusion of US in medical training curricula [1, 2].

Use of US at this level is known as Point-of-Care US (POCUS). However, as pointed out in an editorial in Pediatric Radiology, this is a somewhat equivocal term as it only implies care being provided at the patient level [3]. It would be better to additionally specify who provides POCUS, i.e. a radiologist, a radiological technician or a non-radiologist. Therefore, we will use the term Non-Radiologist POCUS (NR-POCUS). There are several advantages to NR-POCUS: Physicians can gain more specific information in a shorter time, which can directly guide their decisions regarding treatment [4–8]. However, adverse events have also been reported, such as misdiagnosis by insufficiently trained non-radiologists or of clinicians stepping outside the boundaries of NR-POCUS [9].

To gain an overview of NR-POCUS practices in the paediatric setting and to assess their impact in daily practice, we will perform a scoping review. As this is a very broad topic, we will focus our scoping review on abdominal imaging. In addition to summarizing published literature, we aim to identify gaps in the evidence, which can form the basis for future research projects in order to create a firm scientific base for the implementation of NR-POCUS in paediatric medicine.

## Proposed methodology

In this section, we will describe how we will apply each of the scoping study stages identified by Arksey and O’Malley [10] to our study. In contrast to a systematic review, a scoping review addresses the research question in a broader scope. Its aim is to give an overview of the study object and to highlight levels of evidence and gaps in current research.

### Stage 1: Identifying research questions

We have defined the following research questions that will be addressed in this study.

What is known from the literature about the impact of NR-POCUS in the area of abdominal imaging. The focus will be on the following items:

- Provide a state-of-the-art overview of uses of non-radiologist-performed paediatric abdominal point-of-care US.
- Assess how the quality of paediatric NR-POCUS performed by clinicians or clinicians in training in the field of paediatrics and its subdisciplines is evaluated.
- Assess if patient perspective on paediatric US performed by clinicians or clinicians in training in the field of paediatrics and its subdisciplines was evaluated.
- Assess if the financial costs associated with paediatric NR-POCUS performed by clinicians or clinicians in training in the field of paediatrics and its subdisciplines was evaluated.
- Assess if possible harm, following the use of paediatric NR-POCUS performed by clinicians or clinicians in training in the field of paediatrics and its subdisciplines, is described in the literature.

### Stage 2: Identifying relevant studies

The following criteria are defined for studies to be eligible for inclusion in the scoping review. The study must deal with NR-POCUS limited to the abdomen in patients younger than 18 years old. It should describe one of the five topics identified as research questions (stated above). We will not impose any limitations with respect to year or status of publication. Language will be restricted to English only. We will restrict the study to publications involving studies performed in Europe or North America. This is because NR-POCUS in low-resource countries has a different impact on clinical care, as it provides a resource that otherwise would not be available, compared to high resource countries.

Two of the authors (E.A.vW. and R.R.vR.) with help of a clinical librarian (J.G.D.) conceptualised provisional eligibility criteria and exclusion criteria and identified relevant databases to be searched. Eligible studies will be retrieved from peer-reviewed journal articles and main documents and policy statements from relevant professional organisations that have had an impact on this topic, e.g., the American Academy of Pediatrics. Trial searches were run to assess the provisional outcome of our search strategy.

Based on these steps, the following advanced search will be conducted in the following databases: PubMed, Embase and Web of Science Conference Proceedings.

Additionally, we will identify publications by going through the reference lists of articles retrieved through the systematic search. With respect to main documents and policy statements from relevant professional organisations, a manual web-based search will be performed. Finally, electronic poster databases and abstracts of relevant societies (e.g., Society of Pediatric Radiology, European Society of Paediatric Radiology, European Society of Radiology, American College of Emergency Physicians, American Academy of Pediatrics) will be hand searched by E.A.vW. and R.R.vR. for relevant presentations.

Screening will consist of the following three steps:

- The publications found in the search will be exported to a reference manager to scan for and remove duplicated items.
- The resulting data set will be exported to Rayyan, a widely used web-based application, for systematic review [11].
- The database will be screened on title and abstract by two reviewers (E.A.vW. and R.R.vR.) to exclude papers that clearly don’t match the inclusion criteria. Any differences between the two reviewers will be resolved by a second joint review of the disputed literature.

### Stage 3: Study selection

For all publications included after the screening stage, full-text articles will be retrieved. The full texts will be screened by two reviewers (E.A.vW. and R.R.vR.). The following exclusion criteria will be applied:

- Full paper not available.
- Publication published in a language other than English.
- Publication describing a non-paediatric population.
- Publication describing the use of NR-POCUS outside of the abdomen.
- Publication describing a POCUS study performed by radiologists.

Any differences between the two reviewers will be resolved by a second joint review of the disputed literature.

### Stage 4: Charting the data

In this stage, relevant information about the study content will be extracted. This will be achieved via a dedicated data extraction form designed for this review (Table 1). The data extraction will include standard bibliometric information and details of the study characteristics.

**Table 1:** Parameters for data extraction

Bibliometric information	Characteristics of the study	Categories of study characteristics
Study title	Organ of interest	Summary of key message
Authors	Research question/aim(s)	Application to practice
Source/journal	Outcome(s)	Limitations
Year of publication	Key recommendation(s)	Educational/training aspects
Country of origin	Patient population (e.g., diagnosis, age group, etc.)	Legal implications
Discipline of primary author		Cost analyses
Type of study		Safety reporting

We will test the data extraction form on a small number of included studies and, as it will virtually be impossible to design an adequate data extraction form at the outset of this study, we will subsequently permit flexibility in adapting the data extraction form to meet the study’s need.

Although it is not formally a part of the scoping review process, we will assign a level of evidence to all included papers according to the Oxford Centre for Evidence-Based Medicine classification [12].

### Stage 5: Collating, summarising and reporting the data

Our study findings will be presented in a narrative account containing two main aspects. First, a bibliometric analysis describing the nature of the included publications will be provided. Second, the extracted data will be presented in an abdominal organ-based layout. The pros and cons of non-radiologist-performed abdominal POCUS in paediatrics will be provided at an organ-based level.

## Study limitations

As our scoping review is limited to journal articles and main documents and policy statements from relevant professional organisations, we by definition will miss certain publications that might be relevant to the study question.

## Patient and public involvement

Neither patients nor the public were involved in the development of this study design.
